# Biodegradation of polycyclic aromatic hydrocarbons, phenol and sodium sulfate by *Nocardia* species isolated and characterized from Iranian ecosystems

**DOI:** 10.1038/s41598-020-78821-1

**Published:** 2020-12-14

**Authors:** Davood Azadi, Hasan Shojaei

**Affiliations:** 1 Department of Basic and Laboratory Sciences, Khomein University of Medical Sciences, Khomein, Iran; 2 Department of Microbiology, School of Medicine, Isfahan University of Medical Sciences, Isfahan, Iran

**Keywords:** Microbiology, Applied microbiology

## Abstract

Anthropogenic pollutants are known to have adverse effect on ecosystem, biodiversity and human health. Bioremediation is an option that has been widely used to remediate organic contaminants and reduce the risk of these hazardous materials. Microorganisms are readily available to screen and can be rapidly characterized to be applied in many extreme environmental conditions. Actinomycetes have a great potential for the production of bioactive secondary metabolites which have biodegradation activity. This study aimed to screen and characterize *Nocardia* species with biodegradation potential from diverse Iranian ecosystems. The isolates were screened from 90 collected environmental samples, identified and characterized using conventional and molecular microbiological methods including the PCR amplification and sequencing analysis of 16S rRNA and *rpo*B genetic markers. Growth rate in presence of pollutants, chromatography, Gibbs and turbidometric methods were used to determine bioremediation ability. A total of 19 *Nocardia* isolates were recovered from the cultured samples (21.1%) that belonged to 10 various species. The most prevalent *Nocardia* species was *N. farcinica*; 4 isolates (21%), followed by *N. cyriacigeorgica and N. cashijiensis* like; 3 isolates each (15.7%) and *N. asteroides* and *N. kroppenstedtii*; 2 isolates each (10.5%). Our results showed that various *Nocardia* species have great potential for bioremediation purposes, although they have not received much attention of the scholars for such significant usage.

## Introduction

Emerging contaminants due to pharmaceutical and personal care products, plasticizers, synthetic dyes, flame retardants, and pesticides are frequently detected in the environment we live in. In low- and middle-income countries, certain human activities are at the forefront of environmental pollution. Human made changes like overpopulation, pollution, burning fossil fuels, and deforestation have triggered climate change, soil erosion, poor air quality, and undrinkable water. These negative impacts can affect human behavior and can prompt mass migrations or battles over clean water^1,2^.

Removal of emerging contaminants by bioremediation that involves biosorption, bio-uptake, and biotransformation by plants and microorganisms offers numerous advantages such as low-cost and high removal efficiency over other cleanup methods. The key players in bioremediation are microorganisms that are existing everywhere and ideally suited to the task of contaminant destruction and the production of extracellular hydrolytic enzymes that allow them to use environmental contaminants to sustain growth and vital processes^1,3^.

The attractive, generally low-cost bioremediation approach using biodegradation capacity of microorganisms, primarily bacteria, have been considered as an ecological and economical alternative to physicochemical processes to eliminate diffusive contamination of persistent organic pollutants (POPs) in various environments, e.g. soil, sediments and sludge’s. High concentrations of these chemicals act as an environmental stress factor to develop efficient adaptation mechanisms in the adverse environment to metabolize these pollutants^3–5^. Understanding the microbial population and the processes that occur at contaminated sites would be a helpful hint to select and apply the most effective bioremediation method^6,7^. Therefore, the most important step in bioremediation process is isolation and identification of organisms capable of decontamination of pollutants.

Actinomycetes which includes a phylogenetically coherent group i.e., *Corynebacterium*, *Rhodococcus*, *Nocardia*, *Gordonae*, and *Mycobacterium* genera are reported to not only have a high intrinsic resistance to stressful condition but also have potential for degradation of environmental pollutants: members of *Rhodococcus* are able to degrade hydrocarbons, chlorophenols, polychlorinated biphenyls and sulfonated azo dyes^8,9^. Mycobacteria have shown ability to degrade polychlorophenols, heavy metals and diverse polycyclic aromatic hydrocarbons (PAH)^1,9,10^, *Nocardia* have the ability to decompose polycyclic aromatic hydrocarbons (PAH), polychlorinated biphenyls, chlorophenols, sulfonated azo dyes and alkanes^11,12^, and *Gordoniae* can break down alkanes^13^. Although some genera of this family such as *Mycobacterium* and *Nocardia* has a slower growth rate compared to other bioremedial species, yet they are excellent survivors of unfavorable conditions in contaminated environment. Hence, they can successfully compete with fast growing strains such *Pseudomonas* and related bacteria that are well-known for their ability in degradation of hazardous pollutants like aromatic compounds^14^.

Iran is* a* vast country in southwest Asia occupying an area of over 1.64 million square km. This includes an estimation of 8000 species of plants, 535 species of birds, 197 species of mammals and 870 species of fish that indicate biodiversity richness^15^. This great diversity of ecosystems leads to the diversity of microorganisms with distinct enzymatic capabilities. Due to a number of causes, however, Iran is exposed to rapid environmental and biodiversity degradation processes. There is heavy stress on and pollution of scarce environmental resources. Thus, screening and use of microbial species from this group of bacteria with bioremediation activity is a worthwhile effort that can contribute to the development and enhancement of bioremediation technology. The aim of current study was to screen, identify and characterize bioremediation capability of *Nocardia* as one of the most diverse species of actinomycetes with high catabolic capacity from Iranian environmental resources.

## Materials and methods

### Sampling and isolation

**Figure 1:**
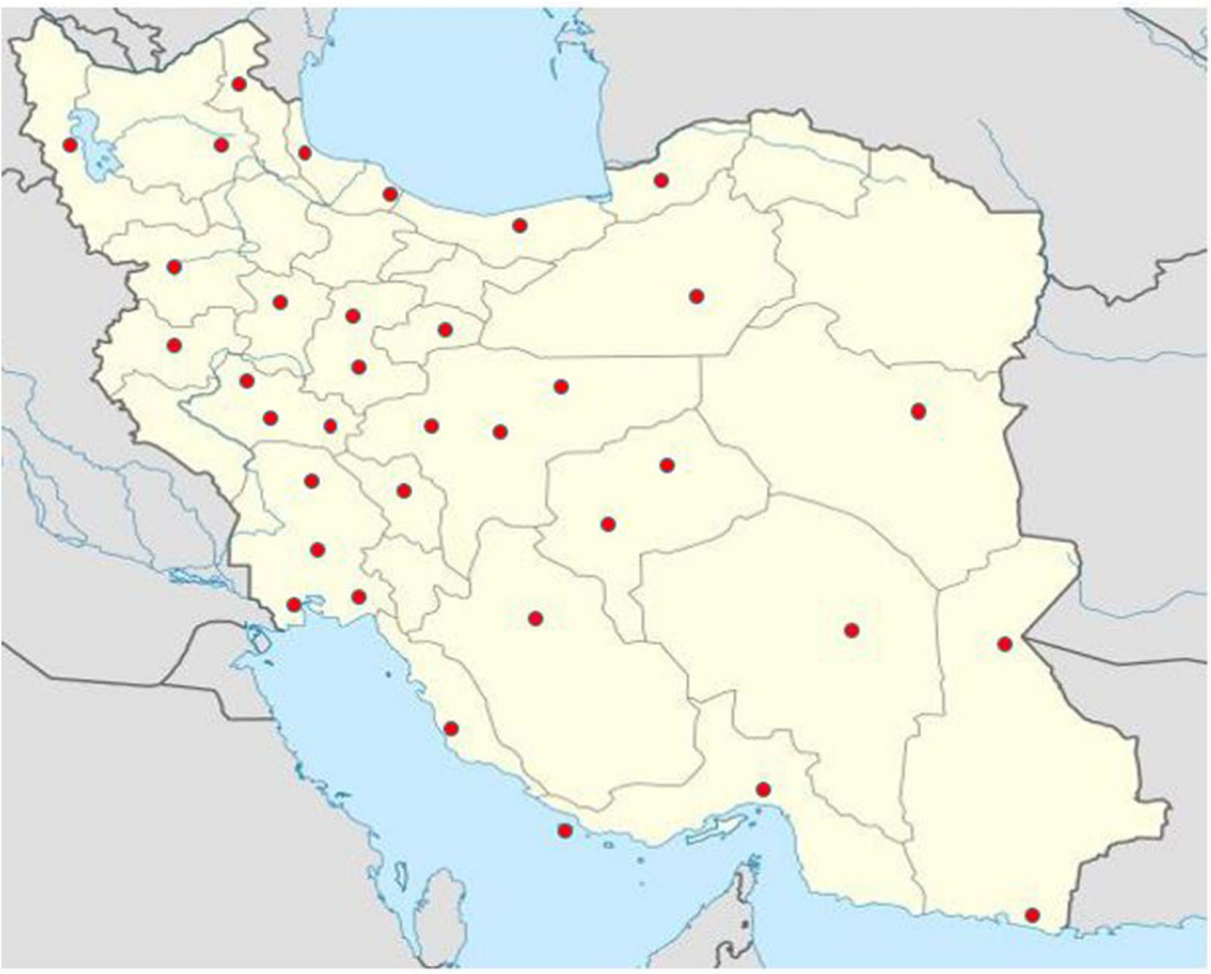
Geographic distribution of sampling sites from Iranian ecosystems. The Figure source is obtained from Google maps and designed by Adobe Photoshop 2017 v18.1.1.252.

Form July 2015 to August 2017, a cross sectional study was carried out on a total number of 90 environmental samples collected from extreme natural and human-related environments such as sea, salt lake and rivers sediments, drinking and non-drinking water, factories and hospital wastewaters, agricultural soil and deserts, forests, oil wells and mines soil (The location and details of the sampling sites are indicated in Fig. 1 and Table 1. The samples were processed based on standard procedures. In summary, for aquatic samples, they were transported at 4 °C to the laboratory and processed within a maximum period of 24 h. The water samples were decontaminated with cetylpyridinium chloride (CPC) 0.005%, for 15 min, and filtered by vacuum through cellulose nitrate filters (0.45 µm, Sartorius, Gottingen, Germany). The filters were rinsed and macerated in tubes containing 15 ml of distilled water. Almost 100 µL aliquots of dissolved samples were transferred into Sauton’s medium, and incubated as described for the soils samples^16^.

**Table 1:** Geographical and physicochemical features of sampling sites from Iranian ecosystems.

Samples	Type of samples	Samples location	Temperature	TDS for water samples	pH	Colony count
AN1	River sediments	Khorramabad	7		6.8	12
AN2	Forest soil	Khorramabad	11		7	18
AN3	Farm soil	Veysian	14		7	6
AN4	River water	Veysian	5	650	7.3	24
AN5	Dam sediments	Dezful	12		7.8	Uncountable
AN6	Dam water	Dezful	13	550	7.6	Uncountable
AN7	Salt lake soil	Arak	4		8	8
AN8	Salt lake water	Arak	7	1224	7.8	8
AN9	Sulfate sodium factory waste water	Arak	8		7.8	40
AN10	Machinery factory waste water	Arak	10		6.3	Uncountable
AN11	Aluminum factory waste water	Arak	14		7.2	16
AN12	Corn farm soil	Shazand	15		7.4	Uncountable
AN13	River sediments	Saman	8	860	7.2	24
AN14	Oil well soil	Omidiyeh	25		6.4	35
AN15	Oil well powder	Omidiyeh	23		6	5
AN16	Crude oil	Omidiyeh	20		6.9	5
AN17	River sediments	Ahwaz	16		7.4	45
AN18	River water	Ahwaz	15	755	7.8	22
AN19	Refinery soil	Abadan	20		7.6	33
AN20	Salt lake soil	Khur and Biabanak	25		8	2
AN21	Orumieh lake sediment	Urmia	26		6.9	8
AN22	Persian gulf water	Kish Island	23	1200	8.2	5
AN23	Persian gulf seaside soil	Kish Island	20		7.9	12
AN24	Persian gulf seaside sediment	Bushehr	24		7.8	22
AN25	Hamoon lake soil	Zabol	30		6.5	33
AN26	Oman sea seaside sediment	Chabahar	18		7.2	8
AN27	Caspian sea soil	Sari	11		6.8	3
AN28	Caspian sea water	Ramsar	10	880	7.3	8
AN29	Tile factory wastewater	Isfahan	24		6.5	50
AN30	Wheat farm soil	Najafabad	23		8.3	25
AN31	Gasoline station soil	Isfahan	8		7.2	33
AN32	Tile factory soil	Najafabad	16		6.1	5
AN33	Tile factory wastewater	Najafabad	26		7/1	5
AN34	Powerhouse soil	Hamadan	11		7	Uncountable
AN35	Corn farm soil	Hamadan	6		8.5	11
AN36	Cement factory soil	Shazand	18		6.3	55
AN37	Powerhouse soil	Arak	20		7.6	32
AN38	Powerhouse soil	Kermanshah	13		8.3	12
AN39	Petrochemicals factory soil	Tabriz	8		7.6	30
AN40	Petrochemicals factory wastewater	Tabriz	5		6.9	5
AN41	Refinery soil	Bandar Abas	18		8.3	42
AN42	Refinery water	Bandar Abas	11	740	7.9	11
AN43	Forest soil	Gorgan	16		7.9	40
AN44	Forest soil	Chaboksar	14		7	Uncountable
AN45	Forest soil	Ramsar	14		7.1	Uncountable
AN46	Bandar Anzali wetlands sediments	Bandar Anzali	20		6.8	50
AN47	Waste incinerator soil	Zarinshahr	18		6.2	Uncountable
AN48	Steel factory soil	Zarinshahr	20		7.2	36
AN49	Sediments of shorabil lake	Ardabil	14		7.8	8
AN50	Water (hot water lake)	Sarein	20	650	7.2	40
AN51	Iron foundry factory soil	Fooladshar	20		7	38
AN52	Petrochemical factory soil	Kermanshah	16		7.2	5
AN53	Cement factory wastewater	Dorud	20		6	20
AN54	Cement factory soil	Dorud	14		6.8	3
AN55	River water	Dorud	14	1200	7.4	22
AN56	Desert soil	Varzaneh	24		8	5
AN57	Textile factory soil	Borujerd	18		7.2	Uncountable
AN58	Textile factory well water	Borujerd	11	480	7.2	10
AN59	Soil of copper mine	Sarcheshmeh	16		8.4	Uncountable
AN60	Water of copper mine	Sarcheshmeh	10	580	7.8	18
AN61	River sediments	Kerman	8		8.2	Uncountable
AN62	River water	Kerman	10	1150	7.5	52
AN63	Desert soil	Yazd	16		7	Uncountable
AN64	Hospital wastewater	Isfahan	8		7.1	36
AN65	Hospital soil	Isfahan	8		6.9	45
AN66	Hospital water	Arak	11	532	7.2	8
AN67	Hospital soil	Tehran	15		7.6	Uncountable
AN68	Hospital wastewater	Tehran	16		6.9	25
AN69	Hospital water	Khorramabad	8	520	7.9	8
AN70	Hospital soil	Khorramabad	12		7.6	Uncountable
AN71	Hospital soil	Yazd	10		7.4	Uncountable
AN72	Hospital wastewater	Kermanshah	8		6.6	16
AN73	Hospital soil	Kermanshah	16		7.1	22
AN74	Hospital wastewater	Ahwaz	11		8.8	18
AN75	Hospital soil	Ahwaz	12		7.6	Uncountable
AN76	Hospital wastewater	Hamadan	8		7.6	16
AN77	Hospital water	Tabriz	10	485	7.2	3
AN78	Hospitals soil	Tabriz	10		7.2	Uncountable
AN79	Hospital soil	Ardabil	14		8.1	6
AN80	Hospital wastewater	Ardabil	9		7.5	28
AN81	Hospital soil	Sari	30		7	18
AN82	Hospital water	Sari	22	830	7.1	5
AN83	Hospital soil	Rasht	28		8.8	Uncountable
AN84	Hospital well water	Rasht	18	640	7.3	14
AN85	Hospital soil	Gorgan	28		8.8	Uncountable
AN86	Hospitals wastewater	Qom	14		7.3	25
AN87	Hospital soil	Qom	9		7	Uncountable
AN88	Hospital wastewater	Birjand	11	510	6.8	3
AN89	Hospital soil	Birjand	10		7.6	20
AN90	Hospital soil	Chabahar	22		7.6	32

For soil samples, 15–30 g soil was taken from 3–5 cm depth of a sampling point at the polluted site and transferred directly to the laboratory. Five grams of soil was transferred to 50 ml sterile centrifuge tube. Then, 20 ml sterile distilled water was added to the tube and vortexed for 5 min, and centrifuged at 4300 RCF at room temperature for 20 min. The pellet and supernatant were decontaminated in separate tubes by 3% sodium lauryl sulfate and 1% NaOH. Afterwards, 100 µL of the decontaminated sample was used to inoculate into the Sauton’s medium which was supplemented with antifungal and antibacterial antibiotics including kanamycin, nystatin and nalidixic acid (each at 50 µg/ml) and were incubated at 25 °C, 30 °C and 35 °C with 5% CO_2_ atmosphere for 3 weeks^17^.

For sediment samples, up to 3 g of samples stirred for 30 min in 100 ml of sterile ringer’s solution (5% v/v). Tenfold serial dilutions of each homogenized suspension was prepared and 200 μl of each of the pretreated 10^−2^, 10^−3^, and 10^−4^ dilutions were inoculated into the Sauton’s medium which was supplemented with antifungal and antibacterial antibiotics including nystatin, kanamycin and nalidixic acid (each at 50 µg/ml). The samples were incubated for 3 weeks at 25 °C, 32 °C and 37 °C with 5% CO_2_ atmosphere^18^.

The details of environmental samples tested during this study are given in Table 2.

**Table 2:** Samples profile, phenotypic and molecular features and bioremediation capability of *Nocardia* isolates from Iranian ecosystems.

No	Sample profile					Biochemical features						16S rRNA analysis			
	Isolates	Location (city)	Source	pH	Temperature	Opt. Tm	Lysozyme Resistance	Pigment	Decomposition of Tyrosine	Decomposition of Xanthene	Decomposition of Hypoxanthine	Similarity (%)^a^	Base pair differences^b^	Identification	Bioremediation capability of
**4**	AN4, AN26,AN27, AN73	Isfahan/Dezful/Yazd	Hospital water& soil/Dam water	8	20	35	+	White	–	–	–	99.78	2/800	*N. farcinica*	Paraffin wax/Crude oil/Phenol
**3**	AN18,AN37,AN47	Khorramabad /Isfahan /Ramsar	River sediment/Forest soil	7–7.2	8–14	35	+	White	–	–	–	100	0/1010	*N. cyriacigeorgica*	PAHs/Thymol
**3**	AN21, AN20, AN30	Isfahan/Arak	Hospital water/Salt Lake sediment	7.6	12	30	+	Yellow	–	–	+	98.92	8/740	*N. cashijiensis* like (sp. nov)	Phenol/Sodium sulfate
**2**	AN31,AN32	Fuladhahr/Dorud	Wastewater/Soil	7–7.4	14–30	35	+	Pink	–	–	–	100	0/847	*N. asteroides*	Crude oil/Natural rubber/Polyethylene/Sodium benzoate
**2**	AN3, AN19	Ahwaz	River sediment	7.6	13	35	+	Yellow	–	–	–	100	0/847	*N. kroppenstedtii*	PAHs/Phenol
**1**	AN7	Arak	Salt lake	6–7.6	20–24	30	+	White	–	–	–	99.5	6/1100	*N. coubleae*	PAHs/Crude oil
**1**	AN35	Abadan	Sea sediment	8	4	35	+	Pink	–	–	–	99.81	3/966	*N. carnea*	Lewisite
**1**	AN5	Kermanshah	Petrochemical factory soil	7	14	35	+	White	–	+	+	100	0/824	*N. otitidiscavarum*	PAHs/Crude oil
**1**	AN22	Shazand	Oil refinery soil	8.5	6	25	+	Pink	+	–	–	99.8	2/1390	*N. fluminea*	PAHs
**1**	AN8	Omidiyeh	Oil well soil	6.4	25	30	+	Pink	–	–	+	99.25	7/939	*N. sungurluensis* like (sp. nov)	Sodium sulfate/PAHs

^a^Similarity; % similarity to the nearest validated species.

^b^Base pair differences: the number of nucleotide differences between the isolates and the nearest validated species.

### Conventional identification

The isolates were initially characterized by conventional phenotypic tests including partial acid-fast staining, growth at 25 °C, 37 °C and 42 °C, pigment production and standard biochemical assays, including resistance to hydrolysis of tyrosine, lysozyme, xanthine, and hypoxanthine tests^19^. The identification was further pursued by molecular testing as follows.

### Molecular identification

Chromosomal DNA from *Nocardia* isolates was extracted using a simple boiling method. In brief, few colonies of bacteria added into 200 ml of TE buffer (Tris EDTA), boiled for 30 min and centrifuged at 10,000 rpm for 10 min. The supernatant was transferred to sterile microtube and centrifuged at 13,000 rpm for 10 min. Precipitated DNA was re-suspended in 50 µl of Milli-Q water and stored at − 20 °C^20^.

The environmental isolates identified phenotypically as *Nocardia* were further verified to the genus level using a specific PCR protocol based on a 596-bp region of the 16S rRNA as recommended by Laurent^21^. For species identification, amplification and direct sequence analysis of 16S rRNA gene was carried out as described by Roth^22^. Sequencing was performed in Bioneer Company (South Korea). The obtained sequences in the current study were aligned manually with all existing sequences of the closely related microorganism retrieved from GenBank database, compared with the relevant sequences and analyzed using the jPhydit program version 1.0^23^.

### Nucleotide sequence accession numbers

The GenBank accession number for the 16S rRNA sequencing of isolated *Nocardia* in this study are listed below.*, N.cyriacigeorgica* (KX685347),* N. coublieae* like *(*KX685350*), N. asteroides (*KX685345*), N. fluminea *like* (*KX685346*), N. otitidiscavarum* (KX685341),* N. kroppenstedii *like (KX685348),* N. cashijiensis *like (KT372140.2), *N. sungurluensis *like (KT372141.1).

### Bioremediation analysis

The sample collection locations have been polluted with crude oil and its derivatives such as PAHs (Polycyclic Aromatic Hydrocarbon), phenol and sodium sulfate. With regard to widespread existence of petroleum resource all around Iran, petrochemical and its derivatives pollutions are the most common environmental pollutants in different region of Iran and have been found in agricultural soil and water, hospital and industrial wastewater and sewages^24–26^. The bioremediation capacity of isolates for these pollutants was evaluated according to Kanali et al.^27^ as follow:

#### Media preparation

To evaluate the bacterial growth in presence of PAH, phenol and sodium sulfate, 100 ml aliquots of Mineral Salt Medium (MSM) were prepared in 250 ml flask as the base media for this experiment. The MSM medium containing (7H20. 0.25-MgSO4, 0.5-KH2PO4, 0.5-K2HPO4, 1-NaCl, 0.009-CaCl2.2H2O, 0.5-KNO3, 0.1-Mn Cl2.4H2O, 0.07-ZnCl2, 0.015-CuCl2.2H2O, 0.025-Ni Cl2.6H2O, 0.12- COCl2.6H2O, 0.025-Na2MO4.2H2O (g/l). The MSM medium then mixed with different substrates as follow.

The media supplemented with 1% PAH mix solution (1–1) purchased from AccuStandard. The PAH mix solution content the following ingredients each at a concentration of 0.2 mg/ml dissolved in dichloromethane and methanol: Acenaphthene, Acenaphthylene, Anthracene, Benzo(b)fluoranthene, 1,2-Benzanthracene, Benzo (a)pyrene, Benzo (k)fluoranthene, Benzo(g,h,i)perylene, Chrysene, Dibenz(a,h)anthracene, Fluoranthene, Fluorene, Indeno(1,2,3-cd) pyrene, Phenanthrene, Naphthalene, Pyrene.

Another set of MSM medium were supplemented with 1% phenol (Merck, Germany) dissolved in deionized water, and the third MSM medium set were supplemented 1% sodium sulfate (Merck, Germany) dissolved in deionized water.

An amount of 1 ml of 0.5 McFarland turbidity (1 × 10^8^ CFU ml^−1^) of the selected isolates was prepared in normal saline, and inoculated into culture media, incubated for 144 h at 30 °C in an orbital shaker (3 RCF). To evaluate the bacterial growth in presence of PAH, phenol or sodium sulfate, samples were collected at 24-h intervals and the optical density of samples measured at a wavelength of 560 nm by spectrophotometer.

Showing sign of growth in the media indicated decomposition and/or consumption of target material by the studied isolates. For final confirmation of degradation, an amount of 5 ml of medium was removed from the flask and examined for the PAH, phenol and sodium sulfate degradation yield according to standard procedures^28–30^ explained as follow.

#### Determination of PAHs phenol and sodium sulfate degradation

After growth, 5 ml of the MSM medium supplemented with PAH was transferred to a screw cap glass tube and supplemented with 0.6 ml of a mixture of tetrachlorethylene and methanol (1:100) as the extraction solvent, vortexed for 10 s, centrifuged at 3000 rpm for 10 min. The organic phase then collected and transferred to a clean tube for further analysis by HPLC. The amount of PAH was measured by injecting 100 µl of the collected organic phase into the HPLC device (Manager 5000, Knauer, Germany), equipped with C18 ultra-sep ES PAH QC specia 6o × 2 mm ID, with water and acetonitrile as mobile phase at a ratio of 5:95, and 0.3 ml min^−1^ flow rate. The adsorption at 254 nm was measured and the PAH content of the sample was calculated using the standard curve and function previously calculated using sterile PAH standards^28^.

Gibbs method was used for determination of phenolic compound^29^. In brief, 5 ml of MSM medium containing phenol which showed bacterial growth was transferred to a sterile tube and the pH was adjusted at 8.0, subjected to centrifugation at 2700*g* for 20 min. 150 µl of the collected supernatant was mixed with 30 µl NaHCO_3_, and 20 µl of Gibbs reagent (2, 6-Dichloroquinone 4-chloroimide) was added to the mixture and shaked for 30 min at 25 °C. The absorbance at 620 nm was recorded and the phenol content determined using the standard curve previously calculated using sterile standards.

From the MSM medium supplemented with sodium sulfate that showed bacterial growth, an amount of 5 ml sample was taken and transferred to a sterile tube. One ml acetic acid (1%) and 1 ml of acetate buffer were added and mixed for 3 min. Afterwards, 1 ml of barium chloride was added and mixed for another 3 min. The turbidity was calculated using spectrophotometry at a wavelength of 420 nm. The amount of consumed sodium sulfate was measured by using the standard curve previously calculated using sterile sodium sulfate standards.

Experiments were conducted in duplicate and average values were computed. The Michaelis-Menten equation was used to compute the in-solution tested materials bio sorption efficiency for each material by each isolate; the results were expressed in percentage terms.

## Results

### Isolation and characterization of *Nocardia* strains

The recorded temperature and pH of the soil samples were in the range of 6 °C to 32 °C and 6.2 to 7.8 respectively. For wastewater and sediment samples, these measures were in the range of 5 °C to 28 °C and 6.8 to 8.2, respectively. The corresponding figures for water samples were 4 °C to 29 °C and 6.4 to 8.0, respectively, and the total dissolved solids (TDS) for the water samples ranged between 560 to 1340 mg/L. The details of soil, water, wastewater and sediment samples and the isolates key properties are presented in Table 1.

From 90, wastewater, sediment, soil, and water samples 19 isolates were identified as *Nocardia* based on phenotypic and biochemical properties, including partial acid-fast staining, pigmentation, resistance to lysozyme and decomposition of xanthine and tyrosine. The presence of a 16S rRNA amplicon of 596 bp in size was a signature band for *Nocardia* species (Table 2).

The 16S rRNA gene sequencing of the isolates revealed that all isolates had nucleotide signatures of *Nocardia* at positions 70–98 (A-T), 293–304 (G-T), 307 (C), 328 (T), 614–626 (A-T), 631(G), 661–744 (G-C), 824e876 (T-A), 825–875 (A-T), 843 (C), and 1122–1151 (A-T)^31^.

Based on phenotypic and molecular data, the *Nocardia* isolates in this study belonged to 8 validated species and 2 unknown or potentially novel species.

The most prevalent *Nocardia* species isolated in our study were *N. farcinica*; 4 isolates (21%), *N. cyriacigeorgica* and an unknown *Nocardia* species closely related to *N. cashijiensis*; 3 isolates (15.7%) each, *N. asteroides* and *N. kroppenstedtii* 2 isolates (10.5%) each. Five single isolates belonged to four established species that is *N. coublieae, N. carne, N. fluminea* and *N. otitidiscavarum*, as well as one unknown *Nocardia* species closely related to *N. sungurluensis.*

The almost complete 16S rRNA gene sequences showed that the isolate AN7 had 99.5% similarities (corresponding to 6 base pair nucleotide differences) with *N. coubleae* type strain DQ235688^T^, the isolates AN21, AN20 and AN30 showed 98.92% similarities with *N. cashijiensis* type strain JCM 11508^T^ (corresponding to 8 base pair nucleotide differences) and the isolate AN8 showed 99.25% similarities (corresponding to 7 base pair nucleotide differences) with *N. sungurluensis* type strain CR3272^T^. This degree of nucleotide differences between our isolates and the established aforementioned *Nocardia* species make them the candidate for further inquiry as the novel species.

**Figure 2:**
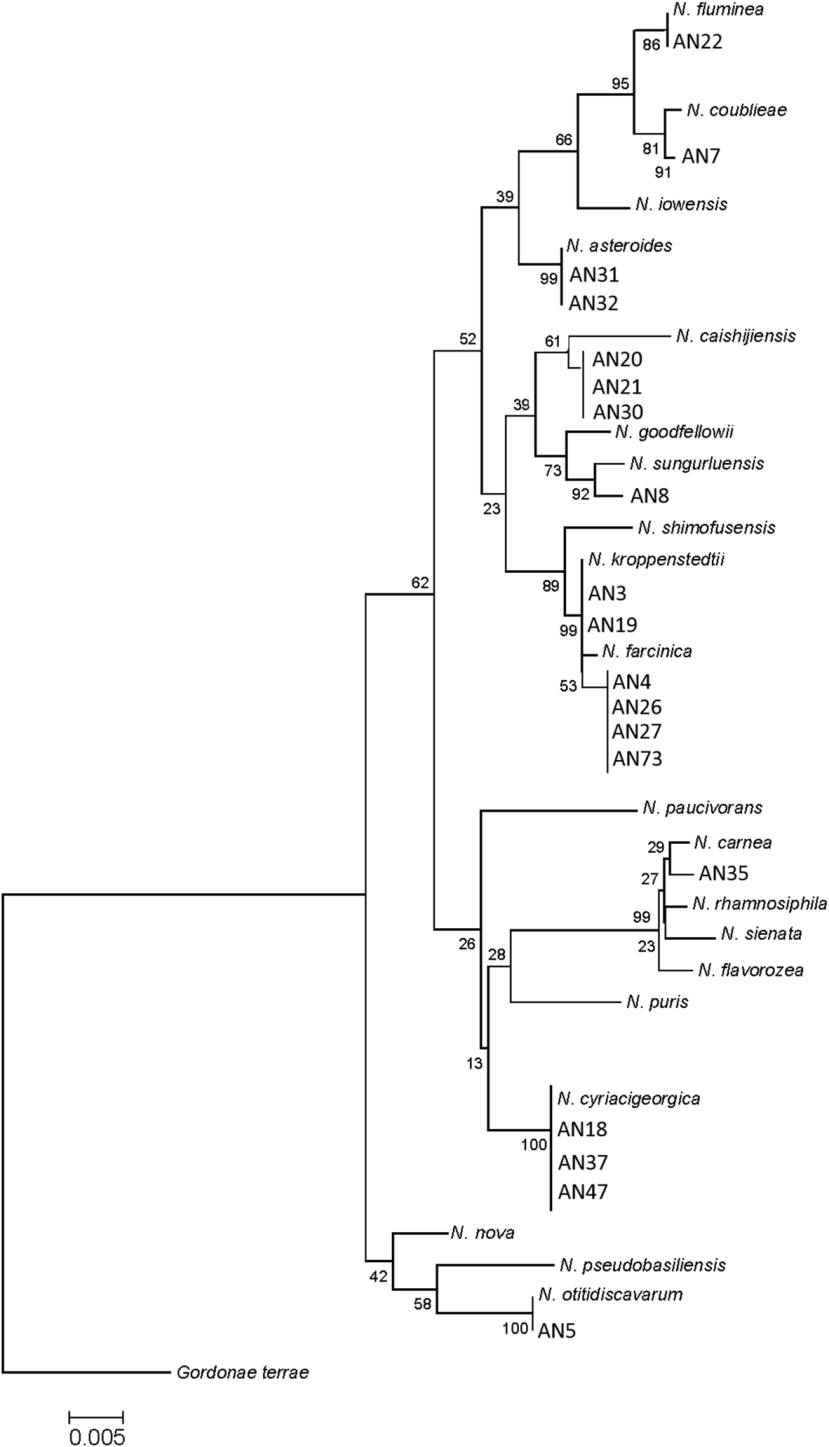
16S rRNA sequence based phylogenetic tree for Iranian biodegrading *Nocardia* isolates and nearest validated species of *Nocardia* depicted by applying MEGA8 software using the neighbor-joining method with bootstrapping values 1000. The figures at each node represent bootstrapping values. The tree was rooted with *G. terrae.*

The relationship between our isolates and the standard established species of *Nocardia* was supported by a high bootstrap value in phylogenetic tree based on 16S rRNA gene depicted by MEGA8. (Fig. 2).

### Bioremediation analysis

With regard to the previous studies the isolated strains were classified into two categories based on the bioremediation ability of the isolates:

1. The isolates that showed bioremediation ability and had been reported previously (Table 1). These included AN4, AN26, AN27 and AN73 which were identified as *N. farcinica*, with capability to degradation of paraffin wax, crude oil and phenol^32,33^. The Isolates AN18, AN37 and AN47 were identified as *N. cyriacigeorgica.* This species has been reported for degradation capacity of PAHs and thymol^34,35^. The isolates AN31and AN32 were identified as *N. asteroides*. This species has been reported for degradation capacity of the crude oil, rubber, polyethylene, sodium benzoate^36–38^. The isolates AN7 was identified as *N. coubleae*. This species has been reported for degradation capacity of PAHs and crude oil degradation^39^. The Isolate AN35 identified as *N. carnea.* This species has been reported for degradation capacity of the lewisite^40^. The Isolate AN5 was identified as *N. otitidiscaviarum* This species has been reported for degradation capacity of PAHs and crude oil^41^.
2. This group included the strains with unreported bioremediation capacity, that is, the isolates, AN4, AN26, AN27and AN73 which were identified as *N. farcinica*, the isolate AN22 which was identified as *N. fluminea,* AN4, AN26, AN27and AN73 which were identified as *N. farcinica*, the isolates AN3, AN19 which were identified as *N. kroppenstedtii*, the isolates AN20, AN21 and AN30 which were identified as *N. cashijiensis*, and the isolate AN8 that was identified as *N. sungurluensis,* In other words for the first time their bioremediation capacity, These strains were tested for PAHs, phenol and sodium sulfate biodegradation capacity according the standard procedures and the following results were obtained.

#### Determination of PAHs, phenol and sodium sulfate degradation

In order to determine PAHs degradation capacity of the selected isolates, the growth and growth rate of the isolates were evaluated in MSM medium in presence of PAHs by measuring optical density at 560 nm in the 24 h intervals.

**Figure 3:**
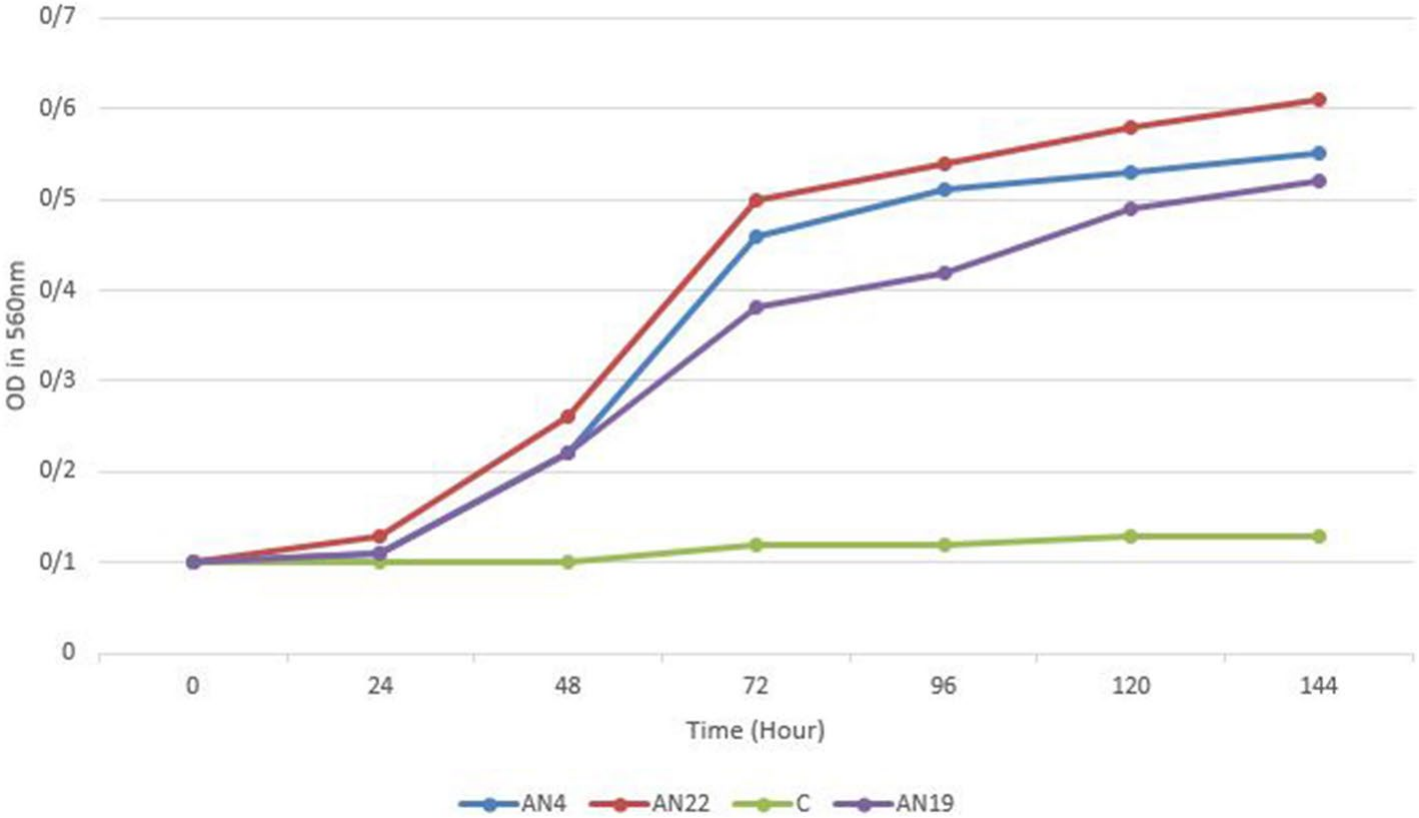
Growth curves of Iranian *Nocardia* isolates over a 24 h. Incubation period at 30 °C in the presence of PAHs. *C* control sample.

As shown in Table 2 the results indicate that, the isolates AN4, AN26, AN27and AN73 which were identified as *N. farcinica*, the isolates AN3, AN19 which were identified as *N. kroppenstedtii* and the isolate AN22 which was identified as *N. fluminea*, were able to consume PAHs compound as an energy and carbon source. These strains were able to degrade one or several compound of the PAH mix. The results also showed that the isolate AN22 which was identified as *N. fluminea* has the highest PAHs degradation rates by degrading more than 90% of the PAHs in the culture medium, followed by the isolates AN4, AN26, AN27 and AN73 were identified as *N. farcinica* and the isolates AN3, AN19 were identified as *N. kroppenstedtii* with 80% and 70% degrading rate of PAHs respectively (Fig. 3). The other isolates, were not able to degrade PAHs.

**Figure 4:**
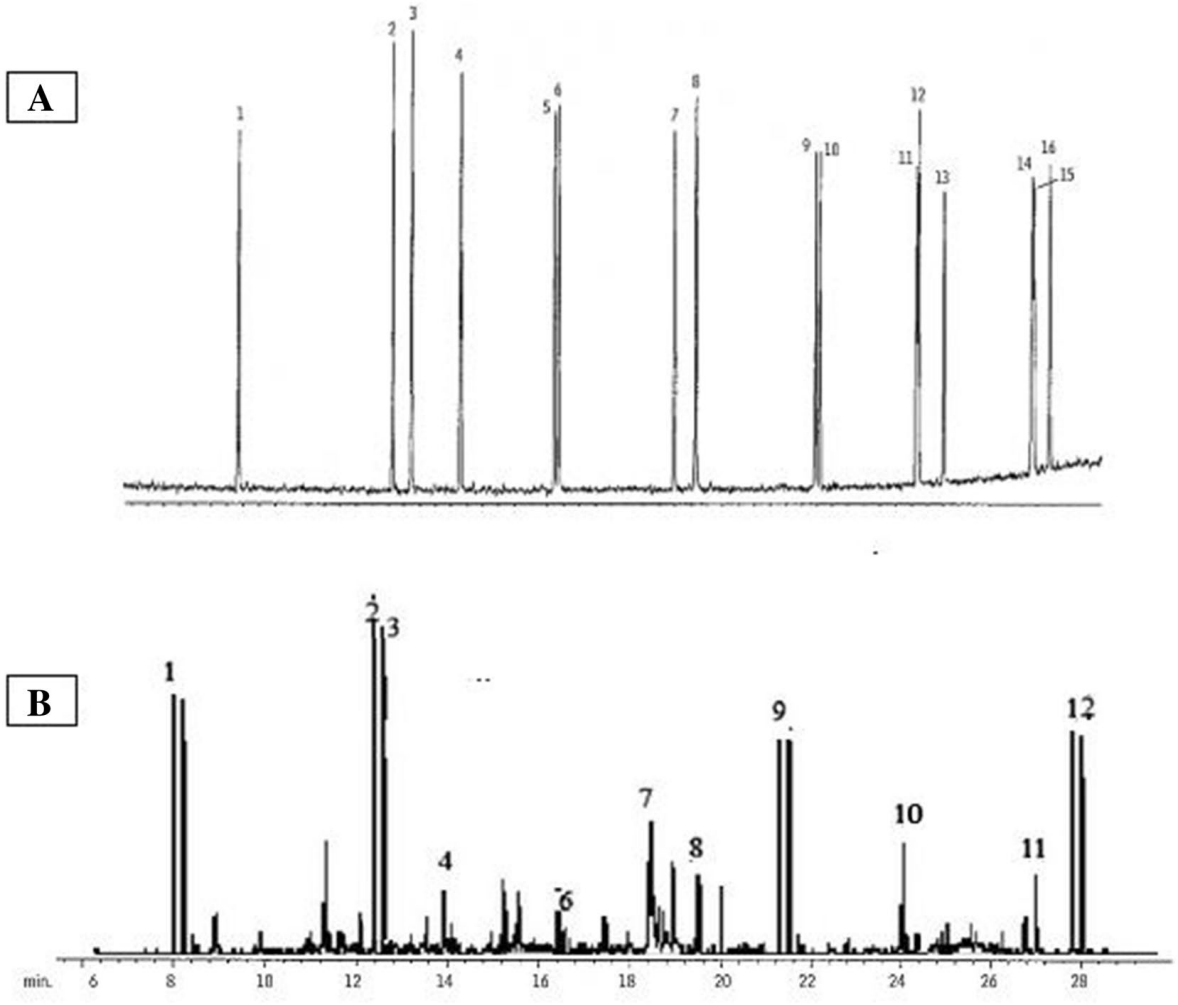
HPLC chromatograms of PAHs mix solution by selected *Nocardia* isolates, (**A**) control samples, (**B**) after 144 h incubation at 30 °C. 1. Naphthalene 2. Acenaphthylene 3. Acenaphthene 4. Fluorene, 5. Phenanthrene, 6. Anthracene, 7. Fluoranthene 8. Pyrene 9. Benzo[a] Anthracene 10. Chrysene 11. Benzo[b]fluoranthene 12. Benzo[k]fluoranthene 13. Benzo[a]pyrene 14. Indeno [1, 2, 3-cd] pyrene 15. Dibenzo [a, h] anthracene.

For final confirmation, the degradation yield and the type of PAH compounds consumed (degraded) by the studied isolates were analyzed by HPLC. The result showed that the isolates AN4, AN26, AN27, AN73, AN3, AN19 and AN22 can degrade different type of PAHs and convert the compounds to other ingredients (Fig. 4).

The phenol biodegradation ability of the isolates was investigated through showing sign of growth in MSM medium supplemented with phenol and by measuring absorbance at 560 nm for samples collected at 24 h intervals.

**Figure 5:**
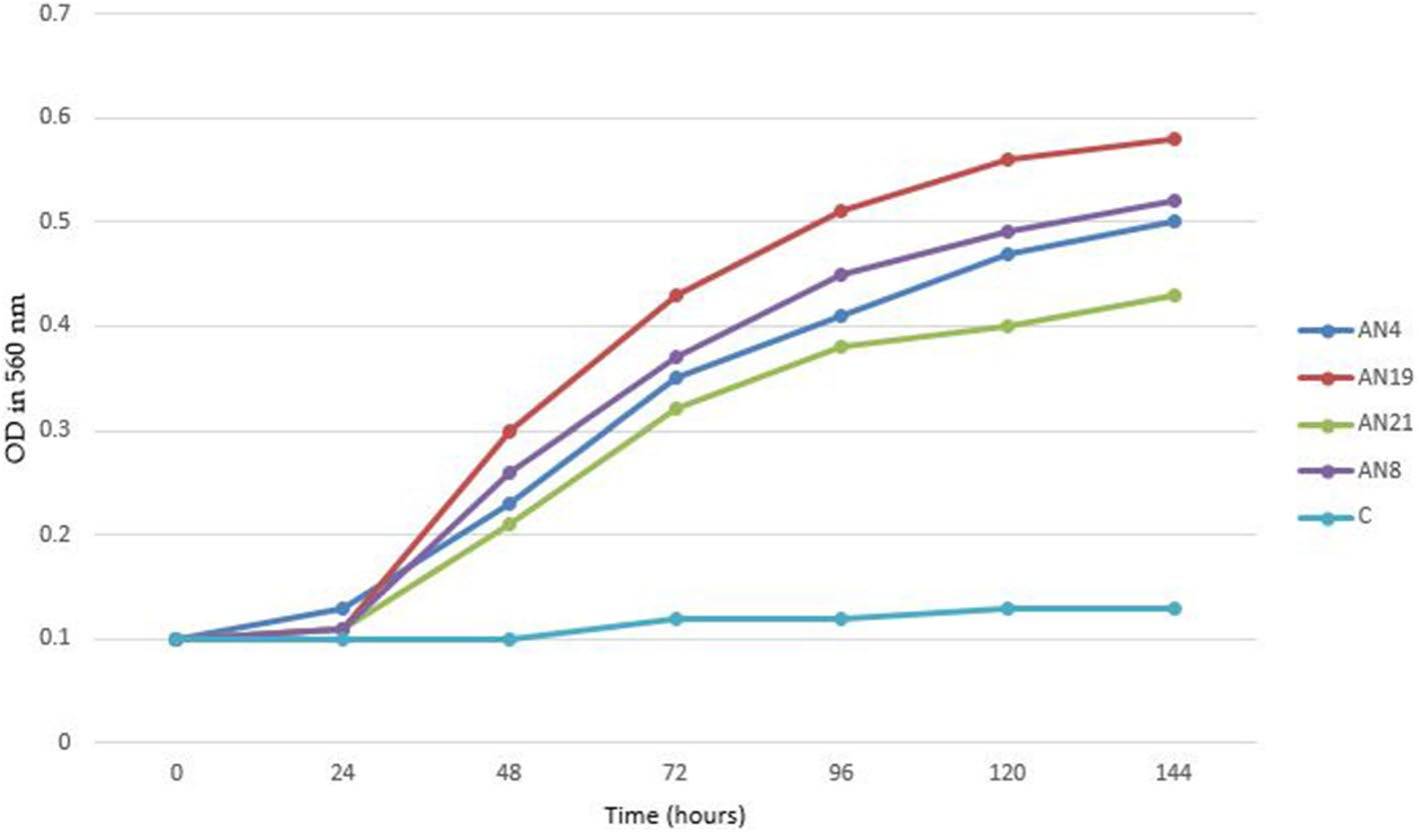
Growth curves of Iranian *Nocardia* isolates over a 24 h. Incubation period at 30 °C in the presence of phenol. *C* control samples.

The results showed that isolates AN4, AN26, AN27 and AN73 which were identified as *N. farcinica*, the isolates AN3 and AN19 which were identified as *N. kroppenstedtii*, the isolates AN20, AN21 and AN30 which were identified as *N. cashijiensis* like and the isolate AN8 that was identified as *N. sungurluensis* like, had ability to grow in the presence of phenol and degrade this compound (Fig. 5).

The results of phenol consumption by the selected isolates showed that isolates AN3 and AN19 that were identified as *N. kroppenstedtii* had the highest phenol degradation rates (95% of phenol degradation in media after 144 h), followed by the *N. sungurluensis* like isolate AN8, *N. farcinica* isolates AN4, AN26, AN27 and AN73 and *N. cashijiensis* isolates AN20, AN21 and AN30 with phenol degradation rate of 85%, 80% and 70% respectively. The other tested isolates were not able to grow significantly in present of phenol in culture media.

Sodium sulfate biodegradation ability of the isolates were investigated by checking for the sign of growth in MSM medium supplemented with sodium sulfate and measuring the optical density at 560 nm. Additionally, in case of growth, the growth rate was evaluated by measuring the cell density via absorbance at 560 nm for samples harvested at 24 h intervals.

**Figure 6:**
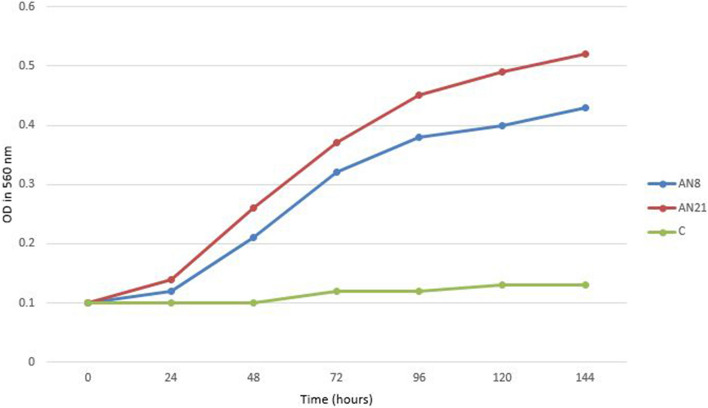
Growth curves of Iranian *Nocardia* isolates over a 24 h. Incubation period at 30 °C in the presence of sodium sulfate. *C* control samples.

The results showed that *N. cashijiensis* like isolates AN20, AN21 and AN30 and *N. sungurluensis* like isolate AN8 had the ability to degrade sodium sulfate (Fig. 6).

The amount of sodium sulfate consumption by the selected isolates was measured by turbidimetry. The results indicated that *N. cashijiensis* isolates AN20, AN21 and AN30 showed highest sodium sulfate biodegradation capability and was able to degrade 100% of the sodium sulfate followed by *N. sungurluensis* isolate AN8 that was able to degrade 85% of the sodium sulfate in prepared medium.

## Discussion

A knowledge of degradation potentials of bacterial strains isolated from polluted sites is pivotal in designing and development of an enduring bioremediation strategy^1,2^. The most of previous studies has focused on isolation of actinomycetes from normal environments which is easily accessible and actinomycetes from these environment can readily be isolated to determine their biological potential. However this is not the case with special environments and actinomycetes that inhabit special environments^42,43^. These special environments are unique due to the extreme conditions that is prevent easy access to them and subsequently actinomycetes from these environment are largely unexplored^8,9^.

Isolation of *Nocardia* species for implementation in bioremediation processes from environmental sources have been a worldwide theme of research^44–46^. In current study, due to their high catabolic capacity as well as high durability in harsh environments such as ecosystems with industrial, domestic and hospital pollution, we studied the process and optimization methods for both isolation and characterization of *Nocardia* species from environmental sources especially those that exist in pristine and untouched ecosystems, as well as the bioremediation capability of the isolates.

In the current study we isolated and characterized 19 biodegrading *Nocardia* strains from 90 samples collected from environmental resources. The isolates belonged to 8 validated species and 2 unknown or potentially novel species. *N. farcinica* was the most encountered isolates. *N. farcinica* was the first historically identified *Nocardia* species and based on the result of current study and previous studies has the ability to degrade the paraffin wax, crude oil and phenol^32,33^. *N. cyriacigeorgica* ranked second that included 16% of the isolates, this organism that was first isolated in 2001 from clinical specimens^47^ has been reported to be able to degrade the PAHs and thymol^34,35^.

In our study *N. asteroides* and *N. kroppenstedtii* ranked the third of the isolates. *N. asteroides* has been reported to have the capacity to degrade the crude oil, rubber, polyethylene and sodium benzoate^36–38^. *N. kroppenstedtii* is an opportunistic pathogen that was first isolated and characterized in 2014 from a patient with a pulmonary infection^48^. However there was no reports on its bioremediation ability. We showed that *N. kroppenstedtii* has the capability to degrade phenol and PAHs.

In the current study, five species that is, *N. coubleae, N. carnea, N. otitidiscavarum, N. fluminea* and an unknown potentially novel *Nocardia* species closely related to *N. sungurluensis* consisted the single isolates. These species were isolated from special ecosystems of Iran including sulfate sodium salt lake, seashore of Persian Gulf, petrochemical factory and farm land around oil well soil respectively.

*Nocardia coubleae* first isolated and characterized in 2007 from oil contaminated soil and determined that has capacity to degrade the PAHs and crude oil^39^. *N. carnea* first characterized in 1913 from clinical samples, and based on the result of previous studies by Nakamiya, determined that this *Nocardia* species has the ability to degrade the lewisite (is an organoarsenic compound use as a chemical weapon, acting as a vesicant and lung irritant)^40^. *N. otitidiscavarum* first isolated and characterized in 1924 from clinical samples^49^. This *Nocardia* species is ubiquitous in environmental resources and has been frequently isolated from the clinical and environmental samples^41,50^. In subsequent studies it was found that *N. otitidiscavarum* has the capacity to degrade PAHs and crude oil^41^. *N. fluminea* first isolated and characterized in 2000 from soil and water^51^, however we found no reports on its bioremediation ability. We showed that this species has the capability to degrade phenol and PAHs in prepared media.

In our study four isolates AN20, AN21 and AN30 and AN8 found to have molecular and phenotypic characteristics different from all previously established *Nocardia* based on 16S rRNA, *hsp*65, and *rpo*B nucleotide sequences*.* These isolates were evaluated for bioremediation activity against PAHs, phenol and sodium sulfate according to standard procedure, and it was determined that the isolates AN21, AN20 and AN30 had a capability to consume and degrade phenol and sodium sulfate compound as a sole carbon and energy source and the isolate AN8 had a capability to degrade sodium sulfate and PAHs. The characterization of these unknown *Nocardia* species remains to be completed using a thorough phenotypic and molecular analysis including cell wall composition analysis and whole genome sequencing.

## Conclusion

Efforts made in the current study have been successful and are supported by evidence from recent reports on the isolation and characterization of novel actinomycetes from poorly researched habitats. Therefore it can be assumed that the screening of untapped regions that possess unexplored actinomycetes will increase the chances of discovering new chemical compounds to be used as a resource for bioremediation significance.

From a biotechnological perspective, the current study showed that the diverse Iranian ecosystems and in particular the nonconventional environments provides vital habitat for actinomycetes and notably *Nocardia* species with capacity for degradation of organic pollutants. This confirms the idea that despite being abundant in environment, *Nocardia* species have been simply ignored for such significant usage. Indeed, there is an untapped potential with regard to bioremedial actinomycetes particularly *Nocardia* that has yet to be discovered and administered in bioremediation of hazardous chemicals.

Normal environments which is easily accessible and actinomycetes isolated from these environment could readily be tapped so that we can analyzed this bacterial species to determine their biological potential. However this is not the case with special environments and actinomycetes that inhabit in these special environments. These special environments are unique due to the extreme conditions that is prevent easy access to them and subsequently actinomycetes from these environment are largely. Thus the group of bacteria from these nascent and unknown habitats are yet to be explored and deserve a special mention.
